# Quality measure and weight loss assessment in patients with type 2 diabetes mellitus treated with canagliflozin or dipeptidyl peptidase-4 inhibitors

**DOI:** 10.1186/s12902-017-0180-8

**Published:** 2017-06-08

**Authors:** Carol H. Wysham, Patrick Lefebvre, Dominic Pilon, Mike Ingham, Marie-Hélène Lafeuille, Bruno Emond, Rhiannon Kamstra, Wing Chow, Michael Pfeifer, Mei Sheng Duh

**Affiliations:** 1 Rockwood Clinic, Spokane, WA USA; 2 Groupe d’analyse, Ltée, 1000 De La Gauchetière West, Bureau 1200, Montréal, Québec H3B 4W5 Canada; 3 Janssen Scientific Affairs, LLC, Raritan, New Jersey USA; 4 Johnson & Johnson, New Brunswick, NJ USA; 5 Analysis Group, Inc., Boston, MA USA

**Keywords:** Canagliflozin, Type 2 diabetes mellitus, Dipeptidyl peptidase-4 inhibitor, HbA1c, Blood pressure, Weight loss

## Abstract

**Background:**

Achieving control of glycated hemoglobin (HbA1c), blood pressure (BP), and body weight (BW) remains a challenge for most patients with type 2 diabetes mellitus (T2DM). In clinical trials, canagliflozin (CANA), an inhibitor of sodium-glucose co-transporter 2, has shown significant improvement compared to some dipeptidyl peptidase-4 (DPP-4) inhibitors in the achievement of such quality measures. This study used recent electronic medical records (EMR) data to assess quality measure achievement of HbA1C, BP, and BW loss in patients treated with CANA versus DPP-4 inhibitors.

**Methods:**

Adult patients with ≥1 T2DM diagnosis and ≥12 months of clinical activity (baseline) before first CANA or DPP-4 prescription (index) were identified in the QuintilesIMS Health Real-World Data EMRs–US database (03/29/2012–10/30/2015). Patients were observed from the index to last encounter. Inverse probability of treatment weighting (IPTW) was used to adjust for observed baseline confounders between groups. Kaplan-Meier (KM) rates and Cox proportional hazard models were used to compare achievement of HbA1c < 7% (among patients <65 years old), HbA1c < 8%, systolic BP < 140 mmHg, diastolic BP < 90 mmHg, and BW loss ≥ 5% among patients not meeting these respective targets at baseline.

**Results:**

A total of 10,702 CANA and 17,679 DPP-4 patients were selected. IPTW resulted in balanced baseline demographic, comorbidity, and disease characteristics (CANA: *N* = 13,793, mean age: 59.0 years; DPP-4: *N* = 14,588, mean age: 58.9 years). Up until 24 months post-index, CANA patients were more likely to reach an HbA1c < 7% (hazard ratio [HR] = 1.10, *P* = 0.007, KM rates: 42.8% vs. 40.3%), an HbA1c < 8% (HR = 1.16, *P* < 0.001, KM rates: 63.7% vs. 60.0%), and a BW loss ≥ 5% (HR = 1.46, P < 0.001, KM rates: 55.2% vs. 46.2%), compared to DPP-4 patients. Up until 12 months post-index, CANA patients were more likely to reach a systolic BP < 140 mmHg (HR = 1.07, *P* = 0.04, KM rates: 87.8% vs. 83.9%). but not a diastolic BP < 90 mmHg (HR = 0.95, *P* = 0.361), compared to DPP-4 patients.

**Conclusions:**

This retrospective study of EMR data covering up to 30 months after CANA approval (March 2013) suggests that patients initiated on CANA were more likely to reach HbA1c, systolic BP, and weight loss objectives specified by general diabetes care guidelines than patients initiated on DPP-4 inhibitors.

## Background

Diabetes is a chronic condition characterized by high glucose levels in the blood. The American Diabetes Association (ADA) estimates that 9.3% of the US population have diabetes in 2012, among which 90–95% had type 2 diabetes mellitus (T2DM) [1]. Diabetes, including T2DM, can lead to several serious and sometimes life-threatening complications including microvascular conditions such as retinopathy and nephropathy, as well as cardiovascular disease [2].

Patients with better glycemic (e.g., glycylated hemoglobin [HbA1c]) and blood pressure (BP) control have better outcomes, including lower risks of complications and better survival, which in turn help to maintain quality of life and reduce complication-related treatment costs [3–5]. Thus, monitoring for and managing high levels of HbA1c and BP as well as body weight (BW) are key components of the standard of care for patients with T2DM [6].

Although personalization of goals is recommended by the Healthcare Effectiveness Data and Information Set (HEDIS) and the ADA, both organizations suggest the following general goals for HbA1c management: HbA1c <7% for nonpregnant adults, and less stringent goals such as HbA1c <8% for those older than 65 or with complications (specifically, “history of severe hypoglycemia, limited life expectancy, advanced microvascular or macrovascular complications, extensive comorbid conditions, or long-standing diabetes”) [6, 7]. The ADA also recommends an objective of BP <140/90 mmHg for all patients [6]. As reported by Bailey et al., various healthcare organizations and community quality improvement programs such as the Centers for Medicare & Medicaid Services: Accountable Care Organization, the Minnesota Community Measurement, the Wisconsin Collaborative for Healthcare Quality, and the Health Collaborative of Greater Cincinnati have established HbA1c goals below 8% and systolic BP <140 mmHg in diabetes patients [8]. Overweight and obesity are highly prevalent among patients with T2DM; [8] furthermore, managing obesity has been shown to have beneficial impacts on T2DM management [6]. Therefore, both the American Association of Clinical Endocrinologists (AACE) and the American College of Endocrinology (ACE) recommend to target a BW loss goal of ≥5% in this population [9].

Various antihyperglycemic agents have been developed to improve glycemic control as well as other vital signs in T2DM patients, as a complement to diet and lifestyle changes [10]. Sodium-glucose co-transporter 2 (SGLT2) inhibitors such as canagliflozin (Invokana® and Invokamet® [CANA]) are one class of agents that work to lower glucose levels by increasing the amount of glucose excreted by the kidneys. Due to their mechanism of action, SGLT2 inhibitors also contribute to reductions in BW and BP [11]. This insulin-independent mechanism of action makes CANA a candidate for add-on therapy with all other antihyperglycemic agents [12]. In recent Phase 3 clinical trials and real-world studies, CANA has been shown to improve HbA1c, BP, and BW in patients with T2DM [13–21].

Another class of antihyperglycemic agents is dipeptidyl peptidase-4 (DPP-4) inhibitors. DPP-4 inhibitors prevent the degradation of incretin hormones by inhibiting the DPP-4 enzyme. Incretin hormones exert their action by stimulation of insulin secretion and inhibition of glucagon release [22]. While DPP-4 inhibitors have been shown to improve HbA1c, only modest improvement in BP and no improvement in BW have been found [23, 24].

In clinical trials comparing CANA 100 mg and CANA 300 mg to sitagliptin 100 mg (a DPP-4 agent) [8, 15, 20], CANA 100 mg was shown to be non-inferior to sitagliptin in achieving diabetes-related quality measure goals for HbA1c, BP, and weight loss at week 52, while CANA 300 mg was shown to be statistically superior. Patients enrolled in these two trials and treated with CANA 100 mg or CANA 300 mg also showed significantly decreased BP and BW as compared to patients treated with sitagliptin.

Few studies have compared glycemic control, BP, and BW loss outcomes between patients initiated on CANA or a DPP-4 agent in a real-world setting [25]. Furthermore, no real-world study has assessed these outcomes over more than one year of follow-up. The aim of the present study was to expand on the clinical benefits mentioned in the clinical trials above and assess achievement of HbA1c, BP, and BW loss goals between a large sample of inadequately controlled patients initiated on CANA versus a DPP-4 agent in a real-world setting where patients can be observed for more than two years following the initiation. In addition, this study assessed the pre- and post-initiation use of antihyperglycemics, antihypertensives, and lipid-lowering agents for patients initiated on CANA versus a DPP-4 agent.

## Methods

### Data Sources

Recent electronic medical records (EMRs) from March 29, 2012 to October 30, 2015 were retrieved from the QuintilesIMS Health Real-World Data Electronic Medical Records – US database (formerly known as Cegedim Strategic Data [CSD] US patient database). This study period was chosen to allow patients initiated on CANA, which was approved by the Federal Drug Administration (FDA) on March 29, 2013 for the treatment of T2DM [26], to have up to one year of medical records prior to CANA initiation while maximizing the length of the observation period following initiation of CANA or DPP-4 treatment. The QuintilesIMS database, in which 40% of contributors are primary care providers and 60% are specialists, includes clinical information and demographic characteristics, such as gender, age, or ethnicity, on more than 35 million patients from all payer types, including Medicare, Medicaid, commercial, and cash. The data are de-identified in compliance with the Health Insurance Portability and Accountability Act (HIPAA) of 1996 to preserve patient confidentiality.

### Study Design and Patient Selection

A retrospective observational study design was used to compare characteristics, attainment of diabetes-related quality measures, and body weight loss between patients treated with CANA or a DPP-4 agent. The index date was defined as the first day with a prescription for CANA or a DPP-4 agent (alogliptin, linagliptin, saxagliptin, or sitagliptin) after March 29, 2013 (the date of CANA approval by the FDA). Patients were classified in the CANA or DPP-4 cohort based on the agent initiated on the index date. Patients were included in the study if they had ≥1 diagnosis for T2DM (ICD-9-CM: 250.x0, 250.x2), ≥12 months of clinical activity prior to the index date (baseline period), ≥1 HbA1c measurement during the baseline period, and if they were 18 years or older at the index date. Moreover, patients initiated on CANA or a DPP-4 agent were excluded if they had a diagnosis for type 1 diabetes mellitus (T1DM, ICD-9-CM: 250.x1, 250.x3) or at least one prescription for a DPP-4 agent during the baseline period. Additionally, patients initiated on CANA and on a DPP-4 agent on the same day were excluded.

The observation period spanned from the index date until the last encounter (i.e., a prescription, a diagnosis, a laboratory test, or a clinical measurement [e.g., vital signs]).

### Outcomes

Demographic and clinical characteristics were analyzed during the 12-month baseline period. Clinical characteristics included the Quan-Charlson Comorbidity Index (Quan-CCI), which was designed to predict a patient’s ten-year mortality risk based on a range of comorbid conditions [27], and the Diabetes Complications Severity Index (DCSI), which was designed to quantify the severity of diabetes complications and help predict the risk of adverse outcomes (for both indexes, a higher score is associated with a higher risk) [28]. Using the Agency for Healthcare Research and Quality (AHRQ) comorbidity definitions, the most common comorbidities were identified [29].

The duration of the index treatment during the observation period (i.e., the number of days between the index date and last day of final prescription for the index treatment) and the daily dose prescribed at the index date were reported. Utilization of antihyperglycemic, lipid-lowering, and antihypertensive agents was also assessed at baseline and during the observation period.

Several goals for diabetes-related quality measures and BW reduction were evaluated during the entire observation period and included HbA1C <7% among patients with a baseline HbA1C ≥7% and aged <65 years old (this goal was also evaluated for all patients with a HbA1C ≥7%), HbA1C <8% among patients with a baseline HbA1C ≥8% and aged ≥65 years old (this goal was also evaluated for all patients with a HbA1C ≥8%), BW loss ≥5%, systolic BP <140 mmHg among patients with a baseline systolic BP ≥140 mmHg, and diastolic BP <90 mmHg among patients with baseline diastolic BP ≥90 mmHg.

### Statistical analysis

For baseline demographic and clinical characteristics as well as treatment patterns, means, standard deviations, and medians were reported for continuous variables; frequencies and percentages were reported for categorical variables. Standardized differences were used to compare baseline characteristics between the two groups and to assess whether cohorts were well-balanced after weighting according to an accepted threshold of ≤10% [30, 31]. Treatment patterns were compared between weighted cohorts using Chi-squared tests for categorical variables and two-sided Student’s t-tests for continuous variables.

To minimize potential confounding and selection bias while retaining a sufficiently large study population, an inverse probability of treatment weighting (IPTW) approach was used [32–34]. First, the propensity score (PS), defined as the probability of being initiated on CANA treatment, was estimated using a multivariate logistic regression model conditional on the baseline covariates (i.e., gender; age categories; ethnicity; region; quarter and year of the index date; use of antihyperglycemic agents at baseline; use of a fixed-dose combination at index date; Quan-Charlson comorbidity index at baseline; number of visits at baseline; closest baseline HbA1c measurement to the index date, closest baseline body mass index [BMI] measurement to the index date, and diagnosis of obesity at baseline). The IPTW approach then used weights derived from PS to create a weighted population that retains all patients and makes both study cohorts similar to the overall study population, such that the distribution of covariates is independent of treatment. Therefore, results obtained with IPTW should be interpreted as the average treatment effect among the overall population. Weights were calculated as the inverse of patients’ estimated probabilities of having their observed initiation treatment (i.e., 1/PS for the CANA group and 1/(1-PS) for the DPP-4 group). Finally, the normalized weights were calculated by dividing each weight by the overall mean weight. After weighting, the sum of weights attributed to each patient in a given cohort may not be equal to the sample size of this cohort; consequently, the effective sample size after weighting may be different than the original sample size (i.e., before weighting). For the analysis of HbA1c among patients aged <65 years, PS and weights were recalculated within this group.

Weighted Kaplan-Meier (KM) survival curves and Cox proportional hazards models (i.e., time-to-event analyses) were used to compare the KM rates and the hazards of reaching diabetes-related goals between the two cohorts. The KM curves of both groups were compared using log-rank tests up to 24 months for all the outcomes and up to 12 months for BP outcomes. The hazards of both cohorts were compared using hazard ratios (HRs), 95% confidence intervals (CIs), and *p*-values which were estimated using weighted Cox models containing a single indicator for treatment cohort.

## Results

### Baseline demographic and clinical characteristics

Among 292,248 patients diagnosed with T2DM, 28,381 met all inclusion and exclusion criteria. Of those, 10,702 were initiated on CANA and 17,679 were initiated on a DPP-4 agent. Most patients in the CANA group were prescribed CANA 100 mg (62.8%) and most patients in the DPP-4 group were prescribed sitagliptin (73.4%), followed by linagliptin (14.3%), saxagliptin (11.2%), and alogliptin (1.8%).

Table 1 presents the baseline demographic and clinical characteristics for the study cohorts. Prior to weighting and relative to patients initiated on DPP-4, patients initiated on CANA were younger (mean age: 57.4 vs. 59.8 years, standardized difference 21.4%), and more likely white (76.9% vs. 74.0%, standardized difference 6.6%). In addition, CANA patients initiated treatment later in the study period, which means that they were also observed and treated for a shorter period of time on average, post-index, compared to patients initiated on a DPP-4 agent. Most patients in both cohorts used other antihyperglycemic (94.2% of CANA patients and 88.7% of DPP-4 patients), lipid-lowering (71.6% of CANA patients and 68.0% of DPP-4 patients), and antihypertensive agents (80.7% of CANA patients and 78.3% of DPP-4 patients) prior to the index date.

**Table 1:** Demographic and clinical characteristics during the 12-month baseline period for unweighted and weighted populations

	Unweighted populations			Weighted populations^a^		
	CANA	DPP-4	Standardized difference^b^ (%)	CANA	DPP-4	Standardized difference^b^ (%)
	*N* = 10,702	*N* = 17,679		*N* = 13,793	*N* = 14,588	
Observation period, months, mean ± SD [median]	10.0 ± 7.4 [8.7]	13.8 ± 8.8 [13.3]	47.3%	11.3 ± 8.2 [10.0]	12.2 ± 8.5 [11.1]	11.3%
Year of index date, *n* (%)						
2013	1,367 (12.8)	5,809 (32.9)	49.3%	2,882 (20.9)	3,606 (24.7)	9.1%
2014	4,653 (43.5)	7,319 (41.4)	4.2%	5,642 (40.9)	6,060 (41.5)	1.3%
2015	4,682 (43.7)	4,551 (25.7)	38.5%	5,270 (38.2)	4,922 (33.7)	9.3%
Demographics						
Gender, female, *n* (%)	5,157 (48.2)	8,707 (49.3)	2.1%	6,757 (49.0)	7,156 (49.1)	0.1%
Age, mean ± SD [median]	57.4 ± 10.7 [58.0]	59.8 ± 12.1 [60.0]	21.4%	59.0 ± 11.5 [59.0]	58.9 ± 11.8 [59.0]	1.1%
Age category, *n* (%)						
18–44 years	1,268 (11.8)	1,948 (11.0)	2.6%	1,487 (10.8)	1,655 (11.3)	1.8%
45–64 years	6,703 (62.6)	9,255 (52.4)	20.9%	7,725 (56.1)	8,215 (56.3)	0.6%
65–74 years	2,220 (20.7)	4,445 (25.1)	10.5%	3,328 (24.1)	3,419 (23.4)	1.6%
≥75 years	511 (4.8)	2,031 (11.5)	24.7%	1,253 (9.1)	1,299 (8.9)	0.6%
Race/Ethnicity, *n* (%)						
White	8,226 (76.9)	13,090 (74.0)	6.6%	10,420 (75.5)	10,973 (75.2)	0.8%
Asian	174 (1.6)	379 (2.1)	3.8%	233 (1.7)	281 (1.9)	1.7%
Black or African American	981 (9.2)	1,748 (9.9)	2.5%	1,307 (9.5)	1,398 (9.6)	0.4%
Hispanic or Latino	163 (1.5)	438 (2.5)	6.8%	279 (2.0)	305 (2.1)	0.5%
Other	113 (1.1)	286 (1.6)	4.9%	183 (1.3)	204 (1.4)	0.6%
Unknown	1,045 (9.8)	1,738 (9.8)	0.2%	1,371 (9.9)	1,427 (9.8)	0.5%
US Region, *n* (%)						
Northeast	2,346 (21.9)	4,294 (24.3)	5.6%	3,168 (23.0)	3,355 (23.0)	0.1%
South	4,674 (43.7)	7,360 (41.6)	4.1%	5,756 (41.7)	6,223 (42.7)	1.9%
Midwest	2,227 (20.8)	3,808 (21.5)	1.8%	3,065 (22.2)	3,123 (21.4)	2.0%
West	1,453 (13.6)	2,215 (12.5)	3.1%	1,802 (13.1)	1,885 (12.9)	0.4%
Unknown	2 (0.0)	2(0.0)	0.6%	3 (0.0)	2(0.0)	0.4%
Clinical characteristics						
Use of medications at baseline, *n* (%)^c^						
Antihyperglycemic agents	10,082 (94.2)	15,678 (88.7)	19.9%	12,679 (91.9)	13,250 (90.8)	3.9%
Biguanides	8,606 (80.4)	13,885 (78.5)	4.6%	11,082 (80.3)	11,582 (80.3)	2.4%
Sulfonylurea derivatives	4,371 (40.8)	7,441 (42.1)	2.5%	5,967 (43.3)	6,100 (41.8)	2.9%
Insulins	3,975 (37.1)	2,715 (15.4)	51.1%	3,305 (24.0)	3,517 (24.1)	0.3%
Glucagon-like peptide 1 agonists	2,793 (26.1)	1,842 (10.4)	41.4%	2,371 (17.2)	2,521 (17.3)	0.3%
Thiazolidinediones	1,279 (12.0)	1,657 (9.4)	8.4%	1,468 (10.6)	1,524 (10.4)	0.6%
Other antihyperglycemic agents^d^	774 (7.2)	685 (3.9)	14.7%	763 (5.5)	775 (5.3)	1.0%
Lipid-lowering agents	7,662 (71.6)	12,022 (68.0)	7.8%	9,734 (70.6)	10,152 (69.6)	2.1%
Antihypertensive agents	8,632 (80.7)	13,846 (78.3)	5.8%	11,139 (80.8)	11,600 (79.5)	3.1%
ACE inhibitors	5,244 (49.0)	8,534 (48.3)	1.5%	6,738 (48.8)	7,206 (49.4)	1.1%
Diuretics	4,510 (42.1)	7,218 (40.8)	2.7%	5,801 (42.1)	6,039 (41.4)	1.3%
Beta blockers	3,140 (29.3)	5,489 (31.0)	3.7%	4,174 (30.3)	4,555 (31.2)	2.1%
Angiotensin II receptor antagonists	2,781 (26.0)	4,120 (23.3)	6.2%	3,574 (25.9)	3,456 (23.7)	5.1%
Calcium channel blockers	2,442 (22.8)	4,076 (23.1)	0.6%	3,288 (23.8)	3,380 (23.2)	1.6%
Antiadrenergic antihypertensives	473 (4.4)	801 (4.5)	0.5%	609 (4.4)	689 (4.7)	1.5%
Vasodilators	117 (1.1)	198 (1.1)	0.3%	163 (1.2)	169 (1.2)	0.2%
Direct renin inhibitors	42 (0.4)	35 (0.2)	3.6%	76 (0.6)	31 (0.2)	5.5%
Selective aldosterone receptor antagonists	13 (0.1)	13 (0.1)	1.5%	17 (0.1)	9 (0.1)	1.9%
Agents for pheochromocytoma	0 (0.0)	1 (0.0)	1.1%	0 (0.0)	1 (0.0)	0.9%
Number of baseline antihyperglycemic agents, mean ± SD [median]^c^	2.3 ± 1.3 [2.0]	1.7 ± 1.1 [2.0]	49.0%	2.0 ± 1.2 [2.0]	2.0 ± 1.3 [2.0]	4.5%
Quan-CCI at baseline, mean ± SD [median]^c^	1.4 ± 1.1 [1.0]	1.4 ± 1.1 [1.0]	2.2%	1.5 ± 1.2 [1.0]	1.4 ± 1.1 [1.0]	3.3%
DCSI at baseline, mean ± SD [median]^c^	0.6 ± 1.1 [0.0]	0.6 ± 1.1 [0.0]	1.0%	0.7 ± 1.1 [0.0]	0.7 ± 1.1 [0.0]	0.9%
Most common DCSI complications, *n* (%)^c^						
Neuropathy	2,063 (19.3)	2,618 (14.8)	11.9%	2,443 (17.7)	2,355 (16.1)	4.2%
Cardiovascular complications	1,536 (14.4)	2,879 (16.3)	5.4%	2,139 (15.5)	2,324 (15.9)	1.2%
Nephropathy	760 (7.1)	1,441 (8.2)	4.0%	1,015 (7.4)	1,225 (8.4)	3.9%
Most common AHRQ complications, *n* (%)^c,h,i^						
Hypertension	7,150 (66.8)	11,271 (63.8)	6.4%	9,143 (66.3)	9,536 (65.4)	1.9%
Obesity	3,423 (32.0)	4,035 (22.8)	20.6%	3,698 (26.8)	3,855 (26.4)	0.9%
Hypothyroidism	1,531 (14.3)	2,379 (13.5)	2.5%	1,949 (14.1)	1,972 (13.5)	1.8%
Depression	1,621 (15.1)	2,307 (13.0)	6.0%	1,989 (14.4)	2,047 (14.0)	1.1%
Chronic pulmonary disease	1,377 (12.9)	2,328 (13.2)	0.9%	1,872(13.6)	1,937 (13.3)	0.9%
Deficiency anemias	789 (7.4)	1,398 (7.9)	2.0%	1,122 (8.1)	1,142 (7.8)	1.1%
Family history of diabetes, *n* (%)	1,292 (12.1)	2,198 (12.4)	0.9%	1,521 (11.0)	1,916 (13.1)	6.5%
Number of classes of medications from which at least one drug is used at baseline, mean ± SD [median]^c,e^	10.1 ± 5.3 [9.0]	9.6 ± 5.4 [9.0]	9.6%	10.1 ± 5.3 [9.0]	9.9 ± 5.4 [9.0]	3.7%
Number of visits at baseline, *n* (%)^c^						
0–4 visits	4,774 (44.6)	8,009 (45.3)	1.4%	5,928 (43.0)	6,494 (44.5)	3.1%
5–9 visits	4,027 (37.6)	6,389 (36.1)	3.1%	5,137 (37.2)	5,358 (36.7)	1.1%
10–14 visits	1,228 (11.5)	2,135 (12.1)	1.9%	1,758 (12.7)	1,766 (12.1)	1.9%
≥ 15 visits	673 (6.3)	1,146 (6.5)	0.8%	970 (7.0)	970 (6.7)	1.5%
HbA1c value at baseline^g^, %, mean ± SD [median]^f^	8.4 ± 1.7 [8.1]	8.3 ± 1.7 [7.9]	9.5%	8.4 ± 1.6 [8.0]	8.3 ± 1.7 [8.0]	0.4%
< 7%, n (%)	1,730 (16.2)	3,278 (18.5)	6.3%	2,366 (17.2)	2,564 (17.6)	1.1%
< 8%, *n* (%)	4,872 (45.5)	8,953 (50.6)	10.3%	6,670 (48.4)	7,064 (48.4)	0.1%
> 9%, *n* (%)	3,088 (28.9)	4,398 (24.9)	9.0%	3,658 (26.5)	3,873 (26.5)	0.1%
Systolic BP value at baseline^g^, mmHg, mean ± SD [median]^f^	130.7 ± 15.2 [130.0]	130.3 ± 15.6 [130.0]	2.4%	130.7 ± 15.4 [130.0]	130.3 ± 15.5 [130.0]	2.5%
< 140 mmHg, *n* (%)	7,811 (73.0)	12,987 (73.5)	1.1%	10,052 (72.9)	10,736 (73.6)	1.6%
≥ 140 mmHg, *n* (%)	2,841 (26.5)	4,525 (25.6)	2.2%	3,670 (26.6)	3,738 (25.6)	2.2%
Missing value, *n* (%)	50 (0.5)	167 (0.9)	5.7%	71 (0.5)	113 (0.8)	3.3%
Diastolic BP value at baseline^g^, mmHg, mean ± SD [median]^f^	77.6 ± 9.6 [78.0]	77.2 ± 9.8 [78.0]	4.0%	77.2 ± 9.8 [78.0]	77.3 ± 9.8 [78.0]	0.8%
< 90 mmHg, *n* (%)	9,452 (88.3)	15,632 (88.4)	0.3%	12,221 (88.6)	12,875 (88.3)	1.1%
≥ 90 mmHg, *n* (%)	1,200 (11.2)	1,880 (10.6)	1.9%	1,501 (10.9)	1,600 (11)	0.3%
Missing value, *n* (%)	50 (0.5)	167 (0.9)	5.7%	71 (0.5)	113 (0.8)	3.3%
BMI value at baseline^g^, kg/m^2^, mean ± SD [median]^f^	35.7 ± 6.1 [35.4]	34.1 ± 6.4 [33.5]	26.5%	34.8 ± 6.3 [34.3]	34.7 ± 6.4 [34.2]	1.4%
< 30 kg/m^2^, *n* (%)	2,014 (18.8)	4,935 (27.9)	21.6%	3,388 (24.6)	3,574 (24.5)	0.2%
30 to < 35 kg/m^2^, *n* (%)	2,986 (27.9)	5,044 (28.5)	1.4%	3,881 (28.1)	4,097 (28.1)	0.1%
≥ 35 kg/m^2^, *n* (%)	5,535 (51.7)	7,148 (40.4)	22.8%	6,221 (45.1)	6,557 (44.9)	0.3%
Missing value, *n* (%)	167 (1.6)	552 (3.1)	10.3%	302 (2.2)	361 (2.5)	1.8%
BW value at baseline^g^, mean ± SD [median]^f^	233.9 ± 53.7 [228]	219.3 ± 53.5 [213.2]	27.1%	226.1 ± 53.8 [220]	224.4 ± 54.3 [218]	3.2%
Missing value, *n* (%)	88 (0.8)	269 (1.5)	6.5%	132.7 (1)	178.1 (1.2)	2.5%
eGFR value at baseline^g^, mL/min/1.73 m^2^, mean ± SD [median]^f^	89.0 ± 23.6 [89.0]	85.1 ± 24.1 [84.8]	16.4%	87.2 ± 23.6 [87.0]	85.8 ± 24.2 [85.5]	6.0%
> 60 ml/min/1.73 m^2^, *n* (%)	5,969 (87.7)	9,821 (83.9)	10.9%	7,913 (86.4)	7,930 (84.3)	6.0%
Missing value, *n* (%)	3,898 (36.4)	5,977 (33.8)	5.5%	4,639 (32.1)	5,184 (33.9)	3.7%

*AHRQ* Agency for Healthcare Research and Quality, *BMI* body mass index, *BP* blood pressure, *BW* body weight, *CANA* canagliflozin, *DCSI* Diabetes Complications Severity Index, *DPP-4* Dipeptidyl Peptidase-4, *eGFR* estimated glomerular filtration rate, *Quan-CCI* Quan-Charlson Comorbidity Index

Notes:

^a^Weighted populations were obtained using inverse probability of treatment weighting based on the propensity score of being treated with CANA. The propensity score was estimated using a multivariate logit regression and baseline covariates included age, gender, US region, race/ethnicity, Quan-Charlson comorbidity index, use of fixed-dose combination at index date, number of visits, closest HbA1c measurement to index date, closest BMI measurement to index date, obesity diagnosis, and quarter of the index date. The number of patients reported for weighted populations corresponds to the sum of weights attributed to patients in each cohort. The sum of weights across both cohorts gives the same total number of patients before (10,702 + 17,679 = 28,381) and after weighting (13,793 + 14,588 = 28,381)

^b^For continuous variables, the standardized difference is calculated by dividing the absolute difference in means of the CANA and the DPP-4 cohorts by the pooled standard deviation of both groups. The pooled standard deviation is the square root of the average of the squared standard deviations. For categorical variables with 2 levels, the standardized difference is calculated using the following equation where P is the respective proportion of participants in each group: (PCANA-PDPP-4)/√[p(1-p)], where *p* = (PCANA + PDPP-4)/2

^c^Evaluated during the 12-month baseline period

^d^Other antihyperglycemic agents include alpha-glucosidase inhibitors, amylin analogs, dopamine receptor agonists, meglitinide analogs, and sodium-glucose co-transporter 2 inhibitors

^e^Classes of medications were taken from the Generic Product Identifier (GPI) classification system

^f^Only the closest measurement from the index date is considered. Includes diabetes-related quality measures evaluated at the index date

^g^Only the closest measurement to the index date (occurring on or prior to the index date) was considered

^h^Reference: Elixhauser A, Steiner C, Kruzikas. D. HCUP Comorbidity Software. Healthcare Cost and Utilization Project (HCUP). October 2015. Agency for Healthcare Research and Quality, Rockville, MD. Available from: https://www.hcup-us.ahrq.gov/toolssoftware/comorbidity/comorbidity.jsp#download

^i^Diabetes is not included

Quan-CCI and DCSI scores were equivalent for patients initiated on CANA compared to those of patients initiated on a DPP-4 (mean Quan-CCI: 1.4; mean DCSI: 0.6). Among the most common DCSI and AHRQ complications, neuropathy, depression, and obesity were more likely for patients initiated on CANA, while cardiovascular complications, nephropathy, and anemia were slightly more likely for patients initiated on a DPP-4 agent. Patients initiated on CANA had higher HbA1c values at index (8.4 vs. 8.3%, standardized difference 9.5%) as well as a higher baseline BMI and weight (mean BMI: 35.7 vs. 34.1 kg/m [2], standardized difference 26.5%; mean weight: 233.9 vs. 219.3 lbs, standardized difference 27.1%), compared to patients initiated on a DPP-4 agent. Baseline characteristics of the IPTW-weighted CANA and DPP-4 cohorts (CANA: *N* = 13,793; DPP-4: *N* = 14,588) were, overall, well balanced (Table 1).

### Index treatment and medication use during the observation period

After weighting, most CANA patients were prescribed 100 mg of CANA (59.6%) at the index date, and the average treatment duration was 267 days. For DPP-4 patients, the majority of patients were prescribed sitagliptin (73.6%) among which 64.9% were initiated on 100 mg. The average treatment duration for DPP-4 patients was 282 days, longer than that of the CANA cohort (*P* < 0.001). Similarly, the average observation period was shorter for CANA patients compared to DPP-4 patients (343 vs. 372 days, *P* < 0.001).

Fewer patients initiated on CANA added or switched to a new antihyperglicemic agent compared to patients initiated on a DPP-4 agent (30.3 vs. 45.4%, *P* < 0.001; Table 2). Notably, fewer CANA patients added or switched to a new glucagon-like peptide 1 agonist, insulin, biguanide, sulfonylurea, or thiazolidinedione agent during the observation period, compared to patients initiated on a DPP-4 agent (all *P* < 0.001; Table 2). CANA patients were also less likely to add or switch to a new SGLT2 agent. This result was expected, since patients would rather switch to an agent with a different mechanism of action. However, CANA patients were as likely as DPP-4 patients to add or switch to another DPP-4 agent (*P* = 0.100).

**Table 2:** Index medication and use of medications following the index date for unweighted and weighted populations

	Unweighted populations			Weighted populations^a^		
	CANA	DPP-4	*P*-value^b^	CANA	DPP-4	*P*-value^b^
	*N* = 10,702	*N* = 17,679		*N* = 13,793	*N* = 14,588	
Observation period, days, mean ± SD [median]	303 ± 224 [266]	420 ± 268 [404]	<0.001	343 ± 250 [305]	372 ± 259 [338]	<0.001
Duration of treatment, days, mean ± SD [median]	245 ± 199 [200]	314 ± 237 [259]	<0.001	267 ± 218 [211]	282 ± 223 [224]	<0.001
CANA dose prescribed at index date^c^, *n* (%)						
100 mg	6,725 (62.8)	-		8,216 (59.6)	-	
300 mg	3,681 (34.4)	-		3,964 (28.7)	-	
50 mg	230 (2.1)	-		862 (6.3)	-	
with metformin 500 mg	73 (0.7)	-		275 (2.0)	-	
with metformin 1000 mg	159 (1.5)	-		591 (4.3)	-	
150 mg	328 (3.1)	-		1,081 (7.8)	-	
with metformin 500 mg	87 (0.8)	-		334 (2.4)	-	
with metformin 1000 mg	243 (2.3)	-		752 (5.5)	-	
DPP-4 dose prescribed at index date^c^, n (%)						
Alogliptin	-	312 (1.8)		-	238 (1.6)	
6.25 mg	-	1 (0.0)		-	1 (0.0)	
12.5 mg	-	104 (0.6)		-	61 (0.4)	
25 mg	-	211 (1.2)		-	178 (1.2)	
Linagliptin	-	2,530 (14.3)		-	2,187 (15.0)	
2.5 mg	-	266 (1.5)		-	152 (1.0)	
5 mg	-	2,268 (12.8)		-	2,037 (14.0)	
Saxagliptin	-	1,986 (11.2)		-	1,532 (10.5)	
2.5 mg	-	559 (3.2)		-	389 (2.7)	
5 mg	-	1,443 (8.2)		-	1,154 (7.9)	
Sitagliptin	-	12,980 (73.4)		-	10,740 (73.6)	
25 mg	-	461 (2.6)		-	399 (2.7)	
50 mg	-	4,934 (27.9)		-	3,443 (23.6)	
100 mg	-	7,691 (43.5)		-	6,971 (47.8)	
New antihyperglycemic agents^d^, n (%)	2,868 (26.8%)	8,543 (48.3%)	<0.001	4,182 (30.3%)	6,616 (45.4%)	<0.001
Sodium-Glucose Co-Transporter 2 (SGLT-2) Inhibitors	524 (4.9%)	3,720 (21.0%)	<0.001	710 (5.1%)	2,907 (19.9%)	<0.001
Dipeptidyl Peptidase-4 (DPP-4) Inhibitors	980 (9.2%)	2,034 (11.5%)	<0.001	1,565 (11.3%)	1,566 (10.7%)	0.100
Glucagon-like peptide 1 (GLP-1) agonists	564 (5.3%)	1,707 (9.7%)	<0.001	819 (5.9%)	1,291 (8.8%)	<0.001
Insulins	454 (4.2%)	1,818 (10.3%)	<0.001	805 (5.8%)	1,260 (8.6%)	<0.001
Sulfonylureas derivatives	399 (3.7%)	1,808 (10.2%)	<0.001	585 (4.2%)	1,335 (9.2%)	<0.001
Biguanides	280 (2.6%)	975 (5.5%)	<0.001	472 (3.4%)	701 (4.8%)	<0.001
Thiazolidinediones	239 (2.2%)	693 (3.9%)	<0.001	366 (2.7%)	505 (3.5%)	<0.001
Meglitinide Analogues	57 (0.5%)	175 (1.0%)	<0.001	102 (0.7%)	130 (0.9%)	0.152
Other	42 (0.4%)	67 (0.4%)	0.859	47 (0.3%)	56 (0.4%)	0.594
Alpha-Glucosidase Inhibitors	20 (0.2%)	66 (0.4%)	0.006	24 (0.2%)	53 (0.4%)	0.002
Amylin Analogs	5 (0.0%)	5 (0.0%)	0.423	5 (0.0%)	4 (0.0%)	0.713
Dopamine Receptor Agonists	4 (0.0%)	8 (0.0%)	0.755	3 (0.0%)	6 (0.0%)	0.408

*CANA* canagliflozin, *DPP-4* dipeptidyl peptidase-4

Notes:

^a^Weighted populations were obtained using inverse probability of treatment weighting based on the propensity score of being treated with CANA. The propensity score was estimated using a multivariate logit regression and baseline covariates included age, gender, US region, race/ethnicity, Quan-Charlson comorbidity index, use of fixed-dose combination at index date, number of visits, closest HbA1c measurement to index date, closest BMI measurement to index date, obesity diagnosis, and quarter of the index date. The number of patients reported for weighted populations corresponds to the sum of weights attributed to patients in each cohort. The sum of weights across both cohorts gives the same total number of patients before (10,702 + 17,679 = 28,381) and after weighting (13,793 + 14,588 = 28,381)

^b^ *P*-values were estimated using chi-square tests or weighted chi-square tests for categorical variables and t-tests or weighted t-tests for continuous variables

^c^There may be more than one prescription at the index date; therefore, categories are not mutually exclusive

^d^Evaluated on or after the index date

### Goal achievement of diabetes-related quality measures and weight loss

#### HbA1c quality measures

**Fig. 1:**
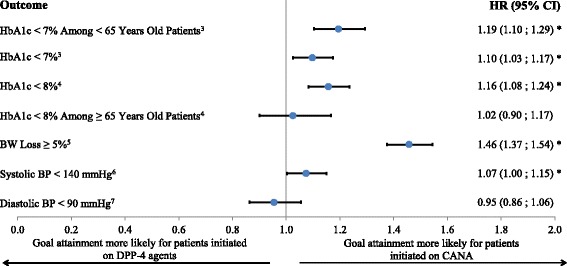
Weighted hazard ratios for HbA1c, weight, and blood pressure outcomes^1,2^. BP: Blood pressure; BW: Body weight; CANA: Canagliflozin; DPP-4: Dipeptidyl-peptidase-4; HR: Hazard ratio. Note: * Significant at the 5% level. 1. Based on the IPTW-weighted population. 2. Estimated using a weighted Cox proportional hazards model containing a single indicator for treatment cohort. 3. Evaluated among patients with a baseline HbA1c ≥7%. 4. Evaluated among patients with a baseline HbA1c ≥8%. 5. Evaluated among patients with a baseline BW measurement. 6. Evaluated among patients with a baseline systolic BP ≥140 mmHg. 7. Evaluated among patients with a baseline diastolic BP ≥90 mmHg

Among patients <65 years old with a baseline HbA1c ≥7% (CANA: *N* = 7,771; DPP-4: *N* = 8,332; mean baseline HbA1c = 8.9% in both cohorts), a higher proportion achieved an HbA1c <7% in the CANA group than in the DPP-4 group at 6, 9, 12, 18 and 24 months post-index (6-month KM rates: CANA vs. DPP-4: 19.6% vs. 16.0%, log rank test *P* < 0.001; 24-month KM rates: CANA vs. DPP-4: 42.2% vs. 38.7%, log rank test *P* < 0.001; Table 3) and CANA patients were 19% more likely to reach an HbA1c <7% compared to DPP-4 patients (HR = 1.19, *P* < 0.001; Fig. 1). Similarly, among all patients with a baseline HbA1c ≥7%, the proportion of patients achieving an HbA1c <7% (CANA: *N* = 11,427; DPP-4: *N* = 12,024; mean baseline HbA1c = 8.8% in both cohorts) was higher in the CANA group than in the DPP-4 group (HR: 1.10, *P* = 0.007; 24-month KM rates: CANA vs. DPP-4: 42.8% vs. 40.3%, log rank test *P* < 0.001, Fig. 1 and Table 3).

**Table 3:** Comparison of weighted kaplan meier rates for HbA1c, weight, and blood pressure outcomes between CANA and DPP-4 cohorts^a^

Outcome	Number of Patients, *n*	Mean Baseline Value		Weighted KM Rates					
				3 Months	6 Months	9 Months	12 Months	18 Months	24 Months
HbA1c < 7% Among < 65 Years Old Patients^b^									
CANA Cohort	7,771	8.92	%	5.67%	19.57%	26.63%	31.39%	38.37%	42.22%
DPP-4 Cohort	8,332	8.93	%	5.31%	16.04%	22.08%	26.49%	33.14%	38.74%
Log-Rank Test				0.364	<0.001*	<0.001*	<0.001*	<0.001*	<0.001*
HbA1c < 8% Among ≥ 65 Years Old Patients^c^									
CANA Cohort	1,951	9.20	%	12.04%	37.52%	47.44%	52.74%	60.77%	63.54%
DPP-4 Cohort	1,980	9.18	%	14.37%	35.35%	45.24%	50.98%	58.93%	65.27%
Log-Rank Test				0.049*	0.511	0.479	0.500	0.416	0.591
HbA1c < 7%^b^									
CANA Cohort	11,427	8.75	%	5.65%	18.46%	25.44%	30.75%	38.21%	42.82%
DPP-4 Cohort	12,024	8.76	%	5.80%	17.18%	23.26%	27.82%	34.67%	40.30%
Log-Rank Test				0.589	0.041*	0.004*	<0.001*	<0.001*	<0.001*
HbA1c < 8%^c^									
CANA Cohort	7,124	9.53	%	12.18%	35.40%	45.25%	50.61%	59.21%	63.69%
DPP-4 Cohort	7,523	9.54	%	12.04%	30.97%	39.55%	44.81%	53.39%	59.98%
Log-Rank Test				0.692	<0.001*	<0.001*	<0.001*	<0.001*	<0.001*
BW Loss ≥ 5%^d^									
CANA Cohort	13,661	226.14	lb	6.24%	18.91%	29.23%	36.69%	47.92%	55.24%
DPP-4 Cohort	14,410	224.41	lb	4.52%	12.46%	19.29%	25.21%	36.36%	46.17%
Log-Rank Test				<0.001*	<0.001*	<0.001*	<0.001*	<0.001*	<0.001*
Systolic BP < 140 mmHg^e^									
CANA Cohort	3,670	150.04	mmHg	40.73%	70.73%	80.89%	87.78%	92.69%	94.34%
DPP-4 Cohort	3,738	150.13	mmHg	41.69%	67.10%	77.78%	83.91%	91.55%	94.53%
Log-Rank Test				0.233	0.056	0.022*	0.005*	0.007*	0.009*
Diastolic BP < 90 mmHg^f^									
CANA Cohort	1,501	94.26	mmHg	47.02%	72.07%	83.84%	89.57%	94.12%	94.78%
DPP-4 Cohort	1,600	94.25	mmHg	49.85%	74.34%	84.00%	89.67%	94.89%	98.14%
Log-Rank Test				0.087	0.141	0.364	0.401	0.373	0.259

*BP* Blood pressure, *BW* Body weight, *CANA* Canagliflozin, *DPP-4* Dipeptidyl-peptidase-4, *KM* Kaplan Meier

Note:

* Significant at the 5% level

^a^Based on the IPTW-weighted population

^b^Evaluated among patients with a baseline HbA1c ≥7%

^c^Evaluated among patients with a baseline HbA1c ≥8%

^d^Evaluated among patients with a baseline BW measurement

^e^Evaluated among patients with a baseline systolic BP ≥140 mmHg

^f^Evaluated among patients with a baseline diastolic BP ≥90 mmHg

Among patients ≥65 years old with a baseline HbA1c ≥8% (CANA: *N* = 1,950; DPP-4: *N* = 1,980; mean baseline HbA1c = 9.2% in both cohorts), a similar proportion achieved an HbA1c <8% in the CANA group and in the DPP-4 group at 6, 9, 12, 18 and 24 months post-index (6-month KM rates: CANA vs. DPP-4: 37.5% vs. 35.4%, log rank test *P* = 0.511; 24-month KM rates: CANA vs. DPP-4: 63.5% vs. 65.3%, log rank test *P* = 0.591; Table 3) and CANA patients were 2% more likely to reach an HbA1c <8% compared to DPP-4 patients, although the result was not found to be statistically significant (HR = 1.02, *P* = 0.710; Fig. 1). Among all patients with a baseline HbA1c ≥8%, the proportion of patients achieving an HbA1c <8% (CANA: *N* = 7,124; DPP-4: *N* = 7,523; mean baseline HbA1c = 9.5% in both cohorts) was higher in the CANA group than in the DPP-4 group (HR: 1.16, *P* < 0.001; 24-month KM rates: CANA vs. DPP-4: 63.7% vs. 60.0%, log rank test *P* < 0.001, Fig. 1 and Table 3)

#### Weight loss

The proportion of patients achieving a BW loss ≥5% among those with ≥1 BW measurement at baseline (CANA: *N* = 13,661, mean baseline BW = 226.1 lb; DPP-4: *N* = 14,410, mean baseline BW = 224.4 lb) was higher in the CANA group than in the DPP-4 group starting at 3 months post-index (3-month KM rates: CANA vs. DPP-4: 6.2% vs. 4.5%, log rank test *P* < 0.001, Table 3); over the entire post-index period, CANA patients were 46% more likely to have a weight loss of 5% than DPP-4 patients (*P* < 0.001; Fig. 1).

#### BP quality measures

Starting at 6 months after the index date, the proportion of patients achieving a systolic BP <140 mmHg among those with a baseline systolic BP ≥140 mmHg (CANA: *N* = 3,670, mean baseline systolic BP = 150.0 mmHg; DPP-4: *N* = 3,738, mean baseline systolic BP = 150.1 mmHg) was higher in the CANA group than in the DPP-4 group (6-month KM rates: CANA vs. DPP-4: 70.7% vs. 67.1%, log rank test *P* = 0.056, Table 3). Moreover, over 12 months of observation, CANA patients were more likely to reach a systolic BP <140 mmHg relative to DPP-4 patients (HR = 1.07, *P* = 0.040; Fig. 1). However, among those with a diastolic BP ≥90 mmHg (CANA: *N* = 1,501, mean baseline diastolic BP = 94.3 mmHg; DPP-4: *N* = 1,600, mean baseline diastolic BP = 94.2 mmHg), no differences were found between the two cohorts for the proportion of patients achieving a diastolic BP <90 mmHg (12-month KM rates: CANA vs. DPP-4: 89.6% vs. 89.7%, log rank test *P* = 0.401; HR: 0.95, *P* = 0.361).

## Discussion

In this study of EMRs collected between March 2012 to October 2015 including 28,381 T2DM patients initiated on CANA or DPP-4 agents, patients initiated on CANA were more likely to attain HbA1c measurements of <7% or 8% during the observation period compared to patients initiated on a DPP-4 agent. Patients initiated on CANA were also significantly more likely to achieve BW reduction of at least 5% and reduction of systolic BP below 140 mmHg compared with patients initiated on a DPP-4 agent. Patients initiated on CANA were as likely as patients initiated on DPP-4 agents to reach a diastolic BP lower than 90 mmHg, this result was expected because greater reductions in systolic BP than diastolic BP were observed in clinical trials for CANA [35]. Overall, attainment of diabetes-related quality measure goals was more likely in CANA patients than in DPP-4 patients despite the fact that a higher proportion of DPP-4 patients added or switched to a new antihyperglycemic agent during the follow-up period, which may have improved quality measure attainment among DPP-4 patients and reduced observed differences between cohorts.

These results were obtained using IPTW to account for observable differences in patients’ characteristics at treatment initiation or over the baseline period. Before weighting, many patient demographic and clinical characteristics were found to be significantly different between the CANA and DPP-4 patient cohorts. Compared to patients initiated on DPP-4: CANA patients were younger; had used a higher number of antihyperglycemic agents at baseline; suffered more from neuropathy, but less from nephropathy; were more often obese, with on average almost 15 more pounds in terms of BW; and had higher baseline HbA1c values, but similar systolic BP. Such differences were also observed in other retrospective studies comparing patients initiated on CANA and patients initiated on sitagliptin, the most commonly prescribed DPP-4 agent [36, 37]. In particular, Grabner et al. found that patients initiated on CANA were on average younger compared to patients initiated on sitagliptin, suffered more from neuropathy and obesity, and had a higher initial HbA1c [36]. However, after applying IPTW, the weighted populations of the current study were well balanced.

This study adds to the growing body of literature reporting on comparisons of CANA and DPP-4s in terms of quality measures [8, 15, 20, 25, 37–39]. Of particular interest, two clinical trials assessed the efficacy and safety of CANA versus sitagliptin over 52 weeks, respectively on background antihyperglycemic treatment with metformin [15] and metformin plus sulfonylurea [20]. Significant differences in favor of CANA 100 mg were identified compared to sitagliptin 100 mg at 52 weeks (12 months) in terms of HbA1c < 8%, BP, BMI and BW loss measure achievement, while no difference was found in terms of HbA1c < 7% [39]. When pooling results from both trials, significant differences in favor of CANA 300 mg were found for the same endpoints, including HbA1c < 7%, when compared to sitagliptin 100 mg [39].

In the present study and following weighting to balance characteristics between patient populations, modest but statistically significant differences between all CANA and DPP-4 patients reaching an HbA1c < 7% appeared at 6 months post-index date, and were observed for those with up to 24 months of follow-up (weighted KM rates respectively 42.8% and 40.3% for CANA and DPP-4, *P* < 0.001). Among patients younger than 65, significant differences in the proportion of patients reaching an HbA1c < 7% were also found as early as 6 months after treatment initiation and consistently for up to 24 months (42.2% vs. 38.7% respectively for CANA and DPP-4 patients, *P* < 0.001). Consistent and numerically larger differences were also found as early as 6 months after treatment initiation and up to 24 months in the proportion of patients reaching HbA1c <8% between CANA and DPP-4 cohorts, although no difference was found in the subgroup of patients aged 65 years or older. However, it is important to consider the clinical context of these older patients when interpreting this finding. Specifically, older age is associated with lower eGFR [40], which may impact dosing considerations. Improving renal and other outcomes (as opposed to targeting HbA1c) may also be of higher priority in older patients, particularly given the promising findings reported for SGLT2s and renal outcomes that have been recently reported [41, 42]. Therefore, future investigations may benefit from exploring other clinical endpoints in older sub-populations of patients. Also of note, although SGLT2 inhibition lowers glucose starting on the first day of initiation, differences with DPP-4 agents are not likely to be found prior to 6 months post-index because HbA1c is likely measured at 6-month intervals among patients with stable glycemic control, as per treatment guidelines (3-month intervals for those not meeting treatment goals) [43].

To the best of our knowledge, only a single study has compared changes in HbA1c and proportions of patients achieving HbA1c <8% and <7% among patients with T2DM treated with CANA versus DPP-4 inhibitors, using data from the real-world setting [25]. In particular, Thayer et al identified that among matched CANA and DPP-4 inhibitor cohorts of 2,776 patients each, change in HbA1c was greater among patients in the CANA cohort than for those in the DPP-4 inhibitor cohort (−0.92% vs. −0.63%, *P* < 0.001), and greater percentages of the CANA cohort relative to the DPP-4 inhibitor cohort achieved HbA1c < 7% (35.4% vs. 29.9%, *P* = 0.022) over a 9 month follow-up [25]. The shorter time to reach the endpoint compared to our study can potentially be explained by the relatively younger population (mean age 56 years vs. 58 years in our study). The aim of the present study was to additionally expand on the clinical benefits other than HbA1c and compare achievement BP, and BW loss goals between a large sample of inadequately controlled patients initiated on CANA or a DPP-4 agent in a real-word setting where patients can be observed for more than two years post-index.

This study was subject to some limitations. First, the data used came from EMRs with a physician centric perspective without any link to healthcare claims. As a consequence, there was no direct link to secondary care data which means that information on hospital visits was not collected. Second, this study relied on prescription data without any knowledge of whether the medication was filled and taken as prescribed. Third, although the CANA prescribing information recommends that patients be initiated on a daily dose of 100 mg [44], a minority of patients in the present study had their first CANA prescription for 300 mg. Two possible explanations for this observation could be that patients were actually initiated on 300 mg, which is consistent with some clinical trials, or that they received samples of CANA 100 mg prior to the index date, which would not have been observed in this database. Fourth, although the study took appropriate measures to reduce confounding, there remains a risk of residual confounding due to unmeasured confounders, which is inherent to observational studies. For instance, sociodemographic characteristics such as economic status and type of insurance coverage were not available while waist circumference and serum creatinine were available for less than 1% of patients. Fifth, the current study assessed goal attainment using recommended thresholds from various healthcare organizations. However, not every patient has the same goals based on his/her demographic and clinical profile; therefore personalization of goals remains important. Sixth, 75% of the study population was White; consequently, results from the current study may not be generalizable to other races/ethnicities. Finally, adverse events were not assessed in this study given that our data source does not allow tracking of patients across different providers and does not include direct link to secondary care data such as hospital visits. Authors believe that the evaluation of adverse events would be impacted by this limitation. As a consequence, it was not possible to evaluate the impact that such adverse events may have had on the outcomes observed in this study. Although the findings should be interpreted in the context of these limitations, large observational studies such as the one currently presented provide a rich insight into real-world clinical populations and practices.

## Conclusion

This real-world retrospective study showed that inadequately controlled patients initiated on CANA were more likely to achieve diabetes-related quality measure goals (HbA1c <7%, HbA1c <8%, BW loss ≥5%, and systolic BP <140 mmHg) compared to patients initiated on a DPP-4 agent. These findings suggest that compared to DPP-4 inhibitors, CANA may be a more effective therapeutic option for improving quality measure goals, which is relevant both for patient outcomes as well as for providers and payers, especially given the importance of quality measure achievement on provider evaluation and reimbursement in the US.

## Abbreviations

AACE: American Association of Clinical Endocrinologists
ACE: American College of Endocrinology
ADA: American Diabetes Association
AHRQ: Agency for Healthcare Research and Quality
BMI: Body mass index
BP: Blood pressure
BW: Body weight
CANA: Canagliflozin
CI: Confidence interval
CSD: Cegedim Strategic Data
DCSI: Diabetes Complications Severity Index
DPP-4: Dipeptidyl peptidase-4
EMR: Electronic medical records
FDA: Federal Drug Administration
HbA1c: Glycated hemoglobin
HEDIS: Healthcare Effectiveness Data and Information Set
HIPAA: Health Insurance Portability and Accountability Act
HR: Hazard ratio
IPTW: Inverse probability of treatment weighting
KM: Kaplan-Meier
PS: Propensity score
Quan-CCI: Quan-Charlson Comorbidity Index
SGLT2: Sodium-glucose co-transporter 2
T1DM: Type 1 diabetes mellitus
T2DM: Type 2 diabetes mellitus
